# Higher Serum DHA and Slower Cognitive Decline in Patients with Alzheimer’s Disease: Two-Year Follow-Up

**DOI:** 10.3390/nu14061159

**Published:** 2022-03-09

**Authors:** Che-Sheng Chu, Chi-Fa Hung, Vinoth Kumar Ponnusamy, Kuan-Chieh Chen, Nai-Ching Chen

**Affiliations:** 1 Department of Psychiatry, Kaohsiung Veterans General Hospital, Kaohsiung City 813, Taiwan; youngtzuchi@hotmail.com; 2 Center for Geriatrics and Gerontology, Kaohsiung Veterans General Hospital, Kaohsiung City 813, Taiwan; 3 Non-Invasive Neuromodulation Consortium for Mental Disorders, Society of Psychophysiology, Taipei 114, Taiwan; 4 Graduate Institute of Medicine, College of Medicine, Kaohsiung Medical University (KMU), Kaohsiung City 807, Taiwan; 5 Department of Psychiatric, Kaohsiung Chang Gung Memorial Hospital and Chang Gung University College of Medicine, Kaohsiung City 833, Taiwan; chifa.hung@gmail.com; 6 College of Humanities and Social Sciences, National Pingtung University of Science and Technology, Pingtung 912, Taiwan; 7 Department of Medicinal and Applied Chemistry, Kaohsiung Medical University (KMU), Kaohsiung City 807, Taiwan; kumar@kmu.edu.tw; 8 Research Center for Environmental Medicine, Kaohsiung Medical University (KMU), Kaohsiung City 807, Taiwan; 9 Department of Medical Research, Kaohsiung Medical University Hospital (KMUH), Kaohsiung City 807, Taiwan; 10 Department of Chemistry, National Sun Yat-Sen University (NSYSU), Kaohsiung City 804, Taiwan; 11 Program of Aquatic Science and Technology, College of Hydrosphere Science, National Kaohsiung University of Science and Technology (NKUST), Kaohsiung City 811, Taiwan; 12 College of Medicine, China Medical University, Taichung 406, Taiwan; u105001036@cmu.edu.tw; 13 Department of Neurology, Kaohsiung Chang Gung Memorial Hospital, Kaohsiung City 833, Taiwan

**Keywords:** Alzheimer’s dementia, omega 3, omega 6, DHA, EPA

## Abstract

Omega-3 polyunsaturated fatty acids (PUFAs), especially eicosapentaenoic acid (EPA) and docosahexaenoic acid (DHA) have been associated with slower rates of cognitive decline. We investigated the association between omega-3 PUFAs and cognitive function in patients with Alzheimer’s disease (AD) receiving acetylcholinesterase inhibitors (AChEIs). This was a prospective cohort study using registered data. Patients with AD receiving AChEIs were recruited from 1 May 2016 to 30 April 2019 and were followed up for two years. Their daily diet record and blood concentration of omega-3 PUFAs were analyzed. Multiple linear and binary logistic regression was used to determine the factors associated with cognitive decline (continuous and dichotomized cognitive change). In the research, 129 patients with AD were identified with a mean age of 76.5 ± 6.6. Patients with AD with lower baseline omega-3 PUFAs levels were associated with a higher risk of cognitive decline than those with higher levels (odds ratio [OR] = 1.067, 95% confidence interval [CI]: 1.012, 1.125; *p* = 0.016) after adjustment. Patients with AD with a lower baseline DHA (OR = 1.131, 95% CI: 1.020, 1.254; *p* = 0.020), but not EPA, were associated with a higher risk of cognitive decline. We found that higher Mini-Nutritional Assessment scores (beta = −0.383, 95% CI = −0.182–−0.048, *p* = 0.001) and total fat (beta = −0.248, 95% CI = −0.067–−0.003, *p* = 0.031) were independently associated with slow cognitive decline in patients with AD receiving AChEIs. The baseline blood levels of omega-3 PUFAs were associated with cognitive decline in patients with AD receiving AChEIs. Future randomized controlled trials are needed to clarify whether this association is causal.

## 1. Introduction

The number of cases of Alzheimer’s disease (AD) around the world is rising due to increased life expectancy and decreased birth rates [1]. Most of the available drugs are symptomatic in nature, aiming to temporarily improve cognitive symptoms, such as acetylcholinesterase inhibitors (AChEIs) and N-methyl-D-aspartate (NMDA) receptor antagonists [2]. Therefore, interventions other than drugs are needed to prevent the incidence of the disease or diminish the progression of AD severity.

Polyunsaturated fatty acids (PUFAs), especially omega-3 (eicosapentaenoic acid, EPA, and docosahexaenoic acid, DHA), have attracted great attention for their ability to prevent cognitive decline [3,4] due to the anti-inflammatory [5] and anti- amyloidogenic [6] properties of PUFAs. A number of epidemiologic studies have found an association between decreased risk of AD and greater consumption of fish (mainly containing EPA and DHA) [7,8]. Similarly, longitudinal studies have reported higher blood concentrations of PUFAs and a decreased risk of developing dementia [9,10,11]. However, in clinical aspects, several randomized controlled trials (RCTs) showed no effect of omega-3 in slowing the rate of cognitive decline among patients already diagnosed with dementia [12,13,14,15], although variation in the dosage, duration and particularly the DHA to EPA ratio might influence the outcomes [16].

EPA and DHA have different biological effects. DHA is the major component of PUFAs inside the brain and is essential in neuroplasticity and neuroprotection. On the other hand, EPA is barely found in the brain and is important in balancing inflammation and immune function [17]. The roles of EPA and DHA in cognition require further investigation. For example, one study showed that DHA but not EPA was associated with poor memory and executive function among a nondemented community population [18], but another study showed only EPA as a risk factor for cognitive decline among patients with dementia [19]. It is of high interest to explore the differential association of EPA or DHA in specific populations. Additionally, there are limited epidemiological studies examining the association between PUFAs and the longitudinal trajectory of cognition in patients already diagnosed with AD.

To further contribute to this issue, we used a routine clinical data resource for a two-year follow-up period to examine the association between baseline EPA and DHA blood levels, dietary habits, and cognitive changes in patients with AD receiving AChEIs. As no previous similar studies were conducted, the present study was considered exploratory.

## 2. Materials and Methods

### 2.1. Participants and Ethical Statement

This prospective cohort study used registered data from 1 May 2016 to 30 April 2019 from Chang Gung Memorial Hospital (CGMH). A total of 129 patients (45 men and 84 women) with AD in the Dementia Outpatient Clinic of Kaohsiung CGMH were enrolled in the study. The study was conducted in accordance with the 1964 Declaration of Helsinki and its later amendments or comparable ethical standards. This study was approved by the Chang Gung Medical Foundation Institutional Review Board, and informed consent was obtained from all participants (CGMH-IRB no. 201600385B0).

The study enrolled patients older than 65 years old from the outpatient department who were diagnosed with AD by certified psychologists or well-trained neurologists, based on comprehensive reviews of medical records, laboratory checks, brain images, and cognitive tests. The diagnosis was made based on the International Classification of Diseases, 10th edition (ICD-10) and the Diagnostic and Statistical Manual of Mental Disorders, fourth edition (DSM-IV). All patients with AD were prescribed AChEIs during the study period. Additionally, patients had to obtain a score between 10 and 26 on the Mini-Mental State Examination (MMSE) or achieve a score of 0.5 or 1 on the clinical dementia rating (CDR). We excluded those with a history of stroke, head injury with neurologic sequelae, Parkinson’s disease, brain tumor, moderate or severe dementia, gastrointestinal problems, colorectal cancer or colorectal inflammation disease. Patients and their primary caregivers, which we defined as providing at least 28 h of care per week, who could not provide detailed information on food consumed within a 72-h period were excluded from this study. The other exclusion criteria for enrolled subjects included end-stage renal disease, tumor under hospice care, chronic pain with regular use of painkillers, and hematologic, endocrine, and autoimmune diseases.

### 2.2. Demographic and Neurobehavioral Assessments

All patients underwent a general physical examination and clinical interview. Patients’ clinical data were obtained, including age, sex and comorbidities, such as type 2 diabetes mellitus, hypertension, hyperlipidemia, coronary artery disease, chronic kidney disease and chronic obstructive pulmonary disease, which were defined according to ICD-10.

A trained neuropsychologist administered the neurobehavioral tests, including the Mini-Mental State Examination (MMSE) [20] to assess the patient with AD for their general intellectual function and clinical dementia rating (CDR) [21] and to evaluate the severity of the patient’s cognitive impairment as determined by both the patient and/or their caregiver in the dementia integrated outpatient clinic. All patients with AD were enrolled from 1 May 2016 to 30 April 2017 in Chang Gung Memorial Hospital (CGMH) with a two-year follow-up until 30 April 2019. Each patient received annual MMSE and CDR examinations when enrolled and once per year during the follow-up period; therefore, three time-point evaluations were performed for each patient.

### 2.3. Definition of Cognitive Decline

In Taiwan, National Health Insurance Administration (NHIA) restricts anti-dementia medications (AChEIs or memantine). These can be only prescribed for patients with AD by a certified neurologist or psychiatrist based on the score of the MMSE or CDR. If there is CDR progression or a decline in the MMSE score of more than two over one-year, disease severity worsening is considered, and anti-dementia medications will be stopped by the NHIA. For this reason, in our routine outpatient clinic follow-up, we performed both tests each year to monitor the cognitive change in patients with dementia.

We used two kinds of definition of cognitive decline: (1) we dichotomized patients into a decline group (defined as CDR score worsening) and stable group (unchanged CDR) within the follow-up period; (2) we assessed the cognitive change during the follow-up period by considering the MMSE at the end of the follow-up minus the baseline MMSE. We recruited a total of 157 patients initially. Of those, 127 patients completed the initial analysis, cognitive follow-up and a food record. Subjects without missing data were enrolled for the study analysis.

### 2.4. Blood Collection and Analysis of the Circulating Biochemical Data

Blood samples were collected between 8 a.m. and 10 a.m. after an overnight fast and analyzed by the central laboratory of Kaohsiung CGMH to determine the serum levels of hemoglobin, total cholesterol, triglycerides, glycated hemoglobin (HbA1c), homocysteine, vitamin B12, and folate. The blood samples were centrifuged at 3000 rpm for 10 min to obtain serum samples, and all circulating biochemical markers were immediately analyzed.

Chromatographic separation of omega-3 PUFAs, including α-linolenic acid (ALA), EPA, DHA, and docosapentaenoic acid (DPA), and omega-6 PUFAs including arachidonic acid (AA), linoleic acid (LA), gamma-linolenic acid (GLA), homo-gamma-linolenic acid (DGLA), docosatetraenoic (DTT) and eicosadienoic acid (ED) was performed using a Pursuit Diphenyl (2.0 mm × 50 mm, 3 μm) column (Agilent Technologies, California, USA) by an ultrahigh performance liquid chromatography (UHPLC) system (Nexera-I 2040C 3D, Shimadzu, Japan). The temperature of the separation column was fixed at 35 °C. Mobile phase A was characterized by: 0.005% formic acid (FA)-water, and in mobile phase B, acetonitrile was utilized in gradient elution as follows: (time min/mobile phase B): 0.01–2/30%, 2–5/60%, 5–9/60%, 9–11/30% and 15/stop. The total chromatographic run time was 15 min. The column flow rate was set to 0.3 mL/min, and the sample injection volume to 3 μL. Omega-3 and 6 PUFAs (GLA, AA, EPA, DGLA, DPA, DTT, ALA, ED, DHA and LA) were detected under negative ion mode-based multiple-reaction monitoring (MRM) using an tandem mass spectrometer (LCMS-8045, shimadzu, Kyoto, Japan) electron spray ionization (ESI) based triple quadrupole mass spectrometer. The heat block temperature was kept at 350 °C, and an interface temperature of 400 °C was used. We applied a 3 L/min flow rate of nitrogen gas as nebulizing gas, heating gas-flow rate (nitrogen) of 10 L/min, and drying gas-flow rate of 10 L/min [22]. The analytical method’s sensitivity to the targeted omega fatty acids was obtained between the concentration range from 0.2 to 1.0 ng mL^−1^ (as quantification limits). The analytical method’s accuracy and precision were calculated in terms of the relative recovery and coefficient of variation (CV) from the triplicate analysis (*n =* 3) of spiked samples and real samples, and the achieved relative recoveries ranged from 80–119%, with CV <9.8%. The obtained recovery and precision results represented an acceptable range in the standard clinical analysis [22].

### 2.5. Dietary Assessments

We used the Mini-Nutritional Assessment (MNA) (maximum score: 30) [23] for nutritional status analysis. The patients and their caregivers also received specific dietary counseling on the required dietary record. In this study, we evaluated their diets by encouraging them to complete a three-day diarized diet record (fluids including milk, juice, and soup). A single well-trained dietitian provided oral instructions on how to perform dietary registration and analysis. The dietitian presented them with a sample spoon, cup, plate, and bowl to permit them to report similar sizes and amounts. The dietary intake was recorded for three days by both patients and primary care givers.

We analyzed the macronutrients according to the information on the Taiwan Food and Drug Administration website (https://consumer.fda.gov.tw/Food/TFND.aspx?nodeID = 178, assessed date on 1 April 2019). The daily intake of energy, macronutrients, and micronutrients was calculated as the average of the three-day food records. After this dietary assessment, patients and their caregivers received personalized dietary counseling and recommendations to improve their dietary inconsistencies. However, changes in the patient’s dietary behavior were not assessed thereafter.

### 2.6. Data Analysis

All values are expressed as the mean ± standard deviation. The chi-squared test was used to compare the categorical variables. Nonparametric methods were used when continuous variables were abnormally distributed. The Mann–Whitney U test was used to compare the differences between the two groups. We conducted binary logistic and multiple linear regression with the step-forward method to determine the most significant factors for cognitive decline in patients receiving AChEIs. Variable selection for multiple analyses was made if: (a) a putative risk factor was determined with significant differences, including the total fat from daily diet analysis and omega-3 from blood analysis, between the two groups; (b) the factors were previously reported to be associated with cognition, including age, gender, education, baseline mini-mental state examination, the MNA scores, daily diet analysis (including total calories, protein, carbohydrate, protein, omega-3 and omega-6) and blood analysis (glycated hemoglobin, total cholesterol, triglyceride, vitamin B12, folate acid, and omega-6). In binary logistic regression, we determined the factors associated with cognitive decline among patients with AD classified as decline group (defined as CDR score worsening) versus stable group (unchanged CDR). In model 1, blood concentration of omega-3 (sum of ALA, EPA, DHA, and DPA) was considered as one of the confounders. If model 1 demonstrated omega-3 as a significant factor associated with cognitive decline, we further conducted model 2 by dividing omega-3 into DHA and EPA. Multiple linear regression was used to determine the factors associated with cognitive decline (the MMSE at the end of the follow-up minus the baseline MMSE) in patients with AD. Statistical analysis was performed using the SPSS version 22.0 software package. Statistical significance was set at *p <* 0.05.

## 3. Results

### 3.1. Demographic Characteristics of Patients with AD Receiving AChEIs in Two Groups

During the two-year follow-up period, the patients with AD receiving AChEIs were divided into a decline group (worsening CDR) and stable group (unchanged CDR). The numbers of patients in the decline and stable groups were 42 and 87, respectively. There were no significant differences in age, gender, cognitive test (MMSE and CDR), and systemic diseases between the two groups (*p >* 0.05). Regarding the nutritional status and diet record, there were no significant differences in the majority of factors between the groups, except for higher daily intake of total fat in the stable group compared to the decline group (stable group, 57.4 ± 11.9 vs. decline group, 49.4 ± 11.8; *p* = 0.042) (Table 1).

### 3.2. Laboratory Data of Patients with AD Receiving AChEIs in Two Groups

There was no significant difference in the majority of the factors between the two groups (*p >* 0.05), except for omega-3, DHA, and EPA. Patients with AD with unchanged CDR during the two-year follow-up period (stable group) had significantly higher levels of omega-3, DHA and EPA compared to those with worsening CDR (decline group) (stable group: omega-3, 10.0 ± 13.8; DHA, 5.2 ± 7.1; EPA, 1.2 ± 0.9. Decline group: omega-3, 3.6 ± 7.2; DHA, 1.8 ± 3.6; EPA, 0.4 ± 1.0. All *p <* 0.05) (Table 2).

### 3.3. Association Factors Influencing Cognitive Decline vs. Cognitive Stability in Patients with AD Receiving AChEIs during the Two-Year Follow-Up Period

We put several possible variables into multiple regression analyses. In model 1, with baseline omega-3 considered as a confounding factor, it was a protective factor for cognitive decline (odds ratio [OR]: 1.067, 95% confidence interval [CI]: 1.012–1.125, *p* = 0.016) among patients with AD receiving AChEIs during the two-year follow-up period. In model 2, by dividing omega-3 into DHA and EPA, we found only baseline DHA to be a protective factor for cognitive decline (OR: 1.131, 95% CI: 1.020–1.254, *p* = 0.020) among patients with AD receiving AChEIs (Table 3).

### 3.4. Association Factors Influencing Trajectory of Cognitive Change in Patients with AD Receiving AChEIs during the Two-Year Follow-Up Period

We found that higher MNA scores (beta = −0.383, 95% CI = −0.182–−0.048, *p* = 0.001) and total fat (beta = −0.248, 95% CI = −0.067–−0.003, *p* = 0.031) were independently associated with slow cognitive decline in patients with AD receiving AChEIs. We did not find an association between omega-3 PUFAs and continuous cognitive change defined by the MMSE at the end of the follow-up minus the baseline MMSE (Table 4).

## 4. Discussion

The findings of the present study were derived from routine clinical practice and thus demonstrated validity. First, we found that patients with AD with lower baseline omega-3 levels were associated with a higher risk of cognitive decline than those with higher baseline omega-3 during the two-year follow-up period after adjustment. Second, by dividing omega-3 into DHA and EPA, we found that patients with AD with baseline lower DHA, but not EPA, were associated with a higher risk of cognitive decline after adjustment. Third, we found baselines of the MNA score and total fat, but not PUFAs, were associated with cognitive change. Fourth, no association was found between the daily intake of omega-3, DHA and EPA and the patients’ cognitive decline.

The findings of the association between lower baseline omega-3 blood levels and a higher risk of cognitive decline were in line with previous prospective cohort studies of communities with no dementia [10,11,24], although some studies showed no association [25]. We further investigated the finding of a lower baseline DHA, rather than EPA, and found this was associated with a higher risk of cognitive decline in patients with AD receiving AChEIs. In one study of up to 22,887 individuals, DHA in the blood was associated with a lower risk of AD and dementia [11]. Another study showed that lower DHA, but not EPA, was associated with worsening of memory and executive function [18]. However, one meta-analysis of 10 cohorts of 2280 cognitively impaired subjects demonstrated that EPA, but not DHA, might be a risk factor for cognitive worsening [19]. The individual association of EPA and DHA on cognitive function remain unknown. The levels of EPA and DHA and the EPA/DHA ratio might influence the findings, and further studies are warranted to address this issue.

We found no association between the daily intake of omega-3, DHA and EPA and the cognitive decline among patients with AD receiving AChEIs for a two-year follow-up period. The omega-3 supplement may associate cognitive function depending on the disease status. For example, previous RCTs of omega-3 supplementation showed significant improvement in cognition among patients with mild cognitive impairment (MCI), but not AD [15,26]. One recent RCT of different components of omega-3 supplementation demonstrated no protective effect on cognition, but on some subitems of Alzheimer’s Disease Assessment Scale-Cognitive (ADAS-cog), such as spoken language ability and constructional praxis [12]. Regarding dementia prevention, more consistent evidence from epidemiological studies has shown an association between higher fish or omega-3 intake and lower cognitive decline and risk of dementia [24]. A meta-analysis of 21 cohort studies for a mean follow-up period of 4.5 years found a dose-dependent relationship between higher fish consumption and the risk of AD [27]. Taken together, omega-3 consumption was associated with a lower risk of developing dementia and less likely cognitive decline in the early stage of AD (for example, MCI); however, it seems to provide limited cognitive protection in patients with late-stage AD. Pathological processes behind AD may begin several decades before the onset of cognitive symptoms [28]. Future studies using disease-modifying interventions, such as omega-3, to prevent age-related cognitive decline and the treatment of MCI, are encouraged.

PUFAs play a role in the disease mechanisms associated with AD through several pathways. In animal models, PUFAs promote synaptic plasticity by increasing long-term potentiation, thus modulating synaptic protein expression to stimulate new dendritic spine formation, which is critical for learning and memory [29]. A brain tissue study of AD found DHA expressed neuroprotection by inhibiting tau phosphorylation, thus preventing neurofibrillary tangles’ accumulation [30]. Moreover, DHA and EPA have been found to alter the amyloid precursor protein, leading to a reduction of amyloid-beta production [31]. In addition to amyloidogenesis, neuroinflammation has been demonstrated to play another key role in the pathogenesis of AD [32]. PUFAs have been demonstrated to ameliorate overactive immune reactions. Lin et al. recently demonstrated in an RCT that EPA supplementation reduced C-C motif ligands 4 (CCL-4), one marker of chronic inflammation associated with the AD pathogenesis [33], in patients with AD, suggesting EPA is an effective low-risk dietary intervention to modulate inflammation [12]. Furthermore, n-3 PUFAs also specifically suppress the expression of proinflammatory cytokines (e.g., tumor necrosis factor alpha (TNF-alpha), interleukin-1 beta (IL-1b) or IL-6) and promote neurotrophin production (e.g., brain-derived neurotrophic Factor (BDNF)) [34]. Although preclinical studies demonstrated a positive effect of PUFAs on cognition, several clinical studies did not show promising results of PUFA supplementation on cognition in both communities and cognitively impaired individuals. Future studies adopting adequate dosage of PUFAs, treatment durations and ratios of DHA/EPA are warranted [16].

### Limitations

First, we used the daily intake of PUFAs as a proxy for omega-3 supplementation, which might not be accurate and may be prone to recall bias. However, each participant received person-to-person dietary registration and analysis education by a single, well-trained dietitian. Second, the present study included a relatively small sample size and was conducted over only a two-year follow-up period. Larger and longer follow-up periods are warranted to confirm the present findings. Third, the association of the PUFA intake would have been influenced by the apoE status [35] and vitamin B [36], which were not examined in the present study. Fourth, the present findings were derived from Taiwanese patients with AD receiving AChEIs; therefore, they are difficult to generalize to those without antidementia medications and of other ethnicities. Fifth, the patients received dietary advice at the beginning of the follow-up period, but their change in diet was not measured. This adds an unmeasured bias to the diets of the study participants during the two-year follow-up. Finally, the present results represent an association between omega-3 PUFAs and cognitive function in patients with AD receiving AChEIs. To further explore whether omega-3 PUFAs influence the effects of AChEIs on cognitive function among patients with AD. Future follow-up studies examining baseline omega-3 PUFAs concentrations among patients with AD receiving AChEIs and those without AChEIs are warranted.

## 5. Conclusions

The present study found an association between lower baseline blood levels of omega-3 PUFAs, particularly DHA, and a higher risk of cognitive decline in patients with AD receiving AChEIs. Future randomized controlled trials of PUFA supplementation are needed to clarify whether this association is causal.

## Figures and Tables

**Table 1:** Demographic characteristics of patients with AD receiving AChEIs in two groups.

	Decline Group (*n =* 42)	Stable Group (*n =* 87)	*p* Value
**Age**	78.1 ± 7.4	75.7 ± 6.1	0.053
Gender			0.367
Male, *n* (%)	16 (38%)	29 (33%)	
Female, *n* (%)	26 (62%)	58 (67%)	
**Cognitive test**			
MMSE	15.8 ± 3.9	17.9 ± 6.6	0.358
CDR	0.7 ± 0.4	0.8 ± 0.5	0.126
**Nutritional status and dietary record**			
Mini nutritional assessment	20.59 ± 7.3	22.2 ± 7.1	0.098
Daily intake (diet analysis)			
Total calories (kcal)	1451.3 ± 226.3	1498.7 ± 305.8	0.798
Protein (g)	51.7 ± 9.9	54.7 ± 12.7	0.346
Carbohydrate (g)	200.8 ± 37.9	192.9 ± 50.1	0.307
Total fat (g)	49.4 ± 11.8	57.4 ± 11.9	0.042 *
Calcium (mg)	387.8 ± 157.1	462.5 ± 317.6	0.607
Omega-3 (mg)	2044.9 ± 1658.5	2075.3 ± 802.3	0.375
Omega-6 (mg)	11912.4 ± 4972.7	12112.1 ± 4895.9	0.901
EPA (mg)	210.5 ± 244.5	231.7 ± 429.5	0.858
DHA (mg)	465.3 ± 430.1	480.3 ± 698.7	0.981
**Systemic diseases**			
Hypertension	13 (30.9%)	22 (25.3%)	0.240
T2DM	20 (47.6%)	44 (50.6%)	0.557
Hyperlipidemia	6 (14.3%)	26 (29.9%)	0.051
Coronary artery disease	10 (23.8%)	12 (13.8%)	0.102
Chronic kidney disease	1 (2.4%)	6 (6.9%)	0.284
Chronic obstructive pulmonary disease	1 (2.4%)	0 (0%)	0.318

Values are expressed in mean ± standard deviation; numbers (percentages); * *p <* 0.05 between group. Abbreviations: AChEIs, acetylcholinesterase inhibitors; AD, Alzheimer’s disease; CDR, clinical dementia rating scale; DHA, docosahexaenoic acid; EPA, eicosapentaenoic acid; MMSE, mini-mental state examination; T2DM, type 2 diabetes mellitus.

**Table 2:** Laboratory data of patients with AD receiving AChEIs in two groups.

	Decline Group (*n =* 42)	Stable Group (*n =* 87)	*p* Value
WBC (1000/μL)	6.3 ± 2.6	6.5 ± 2.8	0.725
Hgb (g/dL)	12.3 ± 1.8	12.8 ± 1.8	0.166
Platelets (1000/μL)	219.6 ± 50.7	222.9 ± 70.2	0.759
AST (U/L)	26.2 ± 11.9	24.7 ± 10.1	0.459
ALT (U/L)	23.4 ± 20.7	19.8 ± 10.9	0.283
BUN (mg/dL)	18.0 ± 7.9	17.0 ± 5.8	0.452
Creatinine (mg/dL)	1.1 ± 0.7	0.9 ± 0.3	0.087
T4 (ng/dL)	1.2 ± 0.2	1.2 ± 0.3	0.663
TSH (μIU/mL)	1.6 ± 1.1	2.3 ± 2.2	0.074
Folic acid (ng/mL)	13.4 ± 10.7	11.0 ± 6.3	0.123
Vitamin B12 (pg/mL)	749.4 ± 858.2	832.7 ± 736.2	0.057
Homocysteine (μmole/L)	23.6 ± 85.2	15.4 ± 8.5	0.540
HbA1c %	6.2 ± 0.8	6.2 ± 0.8	0.670
Triglyceride (mg/dL)	142.2 ± 103.0	134.2 ± 68.2	0.609
Total cholesterol (mg/dL)	184.6 ± 39.5	182.8 ± 37.4	0.800
Omega-3 ^a^ (mg/mL)	3.6 ± 7.2	10.0 ± 13.8	0.028 *
Omega-6 ^b^ (mg/mL)	20.2 ± 39.1	43.6 ± 58.6	0.064
DHA (mg/mL)	1.8 ± 3.6	5.2 ± 7.1	0.023 *
EPA (mg/mL)	0.4 ± 1.0	1.2 ± 0.9	0.049 *

Values are expressed in mean ± standard deviation; numbers (percentages); * = *p <* 0.05 between group. Abbreviations: AChEIs, acetylcholinesterase inhibitors; AD, Alzheimer’s disease; ALT, alanine aminotransferase; AST, aspartate aminotransferase; BUN, blood urea nitrogen; HbA1c, glycated hemoglobin; Hgb, hemoglobin; T4, thyroxine; TSH, thyroid-stimulating hormone; WBC, white blood cell. ^a^ Omega-3 PUFAs included α-linolenic acid (ALA), eicosapentaenoic acid (EPA), docosahexaenoic acid (DHA), and docosapentaenoic acid (DPA); ^b^ omega-6 PUFAs included arachidonic acid (AA), linoleic acid (LA), gamma-linolenic acid (GLA), homo-gamma-linolenic acid (DGLA), docosatetraenoic (DTT) and eicosadienoic acid (ED).

**Table 3:** Multiple logistic regression of predisposing factors for cognitive decline vs. cognitive stability in patients with AD receiving AChEIs.

	Odds Ratio	95% Confidence Interval	*p* Value
Model 1			
Omega-3	1.067	1.012–1.125	0.016 *
Constant	0.199		0.000
Model 2			
Step 1			
DHA	1.131	1.020–1.254	0.020 *
Constant	0.202		0.000

Model 1: Blood concentration of omega-3 (sum of α-linolenic acid (ALA), eicosapentaenoic acid (EPA), docosahexaenoic acid (DHA), and docosapentaenoic acid (DPA)) was considered as one of the factors. Model 2: DHA and EPA were put into analysis instead of omega-3. Possible confounding factors included age, gender, education, baseline mini-mental state examination, mini-nutritional assessment scores, daily diet analysis (include total caloric, protein, fat, carbohydrate, DHA, EPA and omega-6) and blood analysis (glycated hemoglobin, total cholesterol, triglyceride, vitamin B12, folate acid, and omega-6); Multiple logistic regression with a step-forward method was conducted. * = *p <* 0.05

**Table 4:** Multiple linear regression of predisposing factors for the trajectory of cognitive decline in patients with AD receiving AChEIs.

	Beta	95% Confidence Interval	*p* Value
Mini-nutritional assessment scores	−0.383	−0.182–−0.048	0.001 *
Total fat	−0.248	−0.067–−0.003	0.031 *
Constant		3.663–8.212	<0.001 *

Adjusted for age, gender, education, baseline mini-mental state examination, mini-nutritional assessment scores, daily diet analysis (include total caloric, protein, fat, carbohydrate and omega-6) and blood analysis (glycated hemoglobin, total cholesterol, triglyceride, vitamin B12, folate acid and omega-6). * = *p <* 0.05

## Data Availability

The data that support the findings of the study are available from the corresponding author upon reasonable request.
